# Complete Genome Segment Sequences of Tomato Chlorotic Spot Virus from Peanut in Haiti

**DOI:** 10.1128/MRA.00306-19

**Published:** 2019-05-02

**Authors:** Raphael O. Adegbola, Robert C. Kemerait, Scott Adkins, Rayapati A. Naidu

**Affiliations:** a Department of Plant Pathology, Irrigated Agriculture Research and Extension Center, Washington State University, Prosser, Washington, USA; b Department of Plant Pathology, The University of Georgia, Tifton, Georgia, USA; c United States Department of Agriculture, Agricultural Research Service, U.S. Horticultural Research Laboratory, Fort Pierce, Florida, USA; Portland State University

## Abstract

Tomato chlorotic spot virus (TCSV) is emerging as a significant constraint to vegetable and legume crops in the Americas. The complete genome sequence of a TCSV isolate naturally infecting peanut (*Arachis hypogea*) in Haiti was determined in the effort to build epidemiological knowledge of the virus.

## ANNOUNCEMENT

Peanut is an economically important crop cultivated worldwide as a source of dietary protein (1). Several viral species in the genus *Tospovirus*, family *Peribunyaviridae* (2), have been reported to infect peanuts in many countries around the world (3–5). Among them, tomato chlorotic spot virus (TCSV) is receiving increased attention recently due to its expanding geographic and host range in the Caribbean and United States (6–8). Recently, we reported for the first time the occurrence of TCSV in peanut in Haiti (9), which adds to the growing list of tospoviruses infecting peanut. Since this finding has practical implications for the peanut industry in the United States, especially the southeastern and southwestern states, which are the major peanut-producing states, the complete sequences of the small-RNA (S-RNA), medium-RNA (M-RNA), and large-RNA (L-RNA) genome segments of TCSV from peanut were determined by a combination of Sanger sequencing and high-throughput sequencing (HTS). The taxonomic relationship of TCSV from Haiti with TCSV isolates from other countries in the Americas was analyzed to examine the genetic diversity and evolutionary aspects of this emerging tospovirus.

Total RNA was extracted from symptomatic leaves with an RNeasy kit (Qiagen Sciences, Inc., Germantown, MD). The cDNA library preparation was carried out with the TruSeq Stranded Total Ribo-Zero rRNA kit (plant) (Illumina, San Diego, CA) and subjected to HTS on an Illumina HiSeq 2500 platform at the Huntsman Cancer Institute (University of Utah, Salt Lake City, UT). After trimming adapters, raw reads were quality filtered to remove reads with a read length below 125 bp and mapped to the peanut genome (GenBank accession numbers NC_029772 and NC_029785) with the default parameters in CLC Genomics Workbench 8.0 (Qiagen Sciences, Inc.). The quality-filtered reads were assembled *de novo* into contigs using the default parameters in CLC Genomics Workbench 8.0. To validate the sequence data from HTS data, individual genomic segments were amplified with segment-specific primers, cloned, sequenced, and assembled as previously described (10).

A total of 44,742,142 quality-filtered reads with an average length of 124.8 bp were obtained. Of these, 2,096,585 reads were mapped to the virus genome, which formed three contigs corresponding to L-RNA, S-RNA, and M-RNA, with an average coverage of 19,730×, 16,546×, and 5,332×, respectively. Comparative sequence analysis showed that the tripartite genome of the TCSV isolate from peanut is 99% identical at the nucleotide level to the reference TCSV isolate from the Dominican Republic (GenBank accession numbers NC_035482 to NC_035484). The L-RNA sequence is 8,873 nucleotides (nt) long and encodes the putative RNA-dependent RNA polymerase (RdRp) in the viral complementary (vc) strand with a predicted molecular weight of 331 kDa. The M-RNA sequence is 4,847 nt long and encodes a 33.94-kDa nonstructural movement protein (NSm) on the viral (v) strand and a 127.68-kDa glycoprotein precursor (G_N_/G_C_) on the vc strand. The S-RNA sequence is 3,311 nt long and encodes the 52.5-kDa nonstructural silencing suppressor (NSs) on the v strand and the 28.5-kDa nucleocapsid protein (N) on the vc strand. The length of the intergenic region in the M-RNA and S-RNA sequences is 346 nt and 886 nt, respectively. The eight conserved nucleotides and complementary sequences present at the 5′ (AGAGCAAU) and 3′ (AUUGCUCU) termini of the L-RNA, M-RNA, and S-RNA sequences are identical to corresponding sequences in other tospoviruses. The complete genome segment sequences of TCSV from peanut will provide leads for inferring the evolutionary history of the virus and will lead to a better understanding of the extrinsic and intrinsic barriers that influence genome segment exchanges between TCSV and other tospoviruses (7).

### Data availability.

The TCSV genome segment sequences in this announcement are publicly available in GenBank under the following accession numbers: MH427861 (L-RNA), MH427862 (M-RNA), and MH427863 (S-RNA). Raw high-throughput sequencing reads were deposited in the Sequence Read Archive under accession number SRX5643916.

## References

[1] Davis JP, Dean LL 2016 Peanut composition, flavor and nutrition, p 289–345. *In* Stalker HT, Wilson RF (ed), Peanuts genetics, processing, and utilization. AOCS monograph series on oilseeds. Academic Press, San Diego, CA.

[2] Maes P, Adkins S, Alkhovsky SV, Avšič-Županc T, Ballinger MJ, Bente DA, Beer M, Bergeron É, Blair CD, Briese T, Buchmeier MJ, Burt FJ, Calisher CH, Charrel RN, Choi IR, Clegg JCS, de la Torre JC, de Lamballerie X, DeRisi JL, Digiaro M, Drebot M, Ebihara H, Elbeaino T, Ergünay K, Fulhorst CF, Garrison AR, Gāo GF, Gonzalez J-PJ, Groschup MH, Günther S, Haenni A-L, Hall RA, Hewson R, Hughes HR, Jain RK, Jonson MG, Junglen S, Klempa B, Klingström J, Kormelink R, Lambert AJ, Langevin SA, Lukashevich IS, Marklewitz M, Martelli GP, Mielke-Ehret N, Mirazimi A, Mühlbach H-P, Naidu R, Nunes MRT, Palacios G, 2019 Taxonomy of the order *Bunyavirales*: second update 2018. Arch Virol 164:927–941. doi:10.1007/s00705-018-04127-3. 30663021 PMC6581445

[3] Golnaraghi AR, Shahraeen N, Pourrahim R, Ghorbani S, Farzadfar S 2001 First report of a tospovirus infection of peanuts in Iran. Plant Dis 85:1286. doi:10.1094/PDIS.2001.85.12.1286C. 30831798

[4] Appiah AS, Offei SK, Tegg RS, Wilson CR 2016 Varietal response to groundnut rosette disease and the first report of *Groundnut ringspot virus* in Ghana. Plant Dis 100:946–952. doi:10.1094/PDIS-07-15-0838-RE. 30686150

[5] Vijayalakshmi G, Haokip BD, Karthikeyan G, Alice D, Ganapathy N, Asokan G, Rajendran L, Kennedy JS 2016 First report of field infection of *Capsicum chlorosis virus* on groundnut (*Arachis hypogaea*) in India (Tamil Nadu). Plant Dis 100:2339. doi:10.1094/PDIS-05-16-0595-PDN.

[6] Batuman O, Rojas MR, Almanzar A, Gilbertson RL 2014 First report of *Tomato chlorotic spot virus* in processing tomatoes in the Dominican Republic. Plant Dis 98:286. doi:10.1094/PDIS-07-13-0685-PDN. 30708759

[7] Webster CG, Frantz G, Reitz SR, Funderburk JE, Mellinger HC, McAvoy E, Turechek WW, Marshall SH, Tantiwanich Y, McGrath MT, Daughtrey ML, Adkins S 2015 Emergence of *Groundnut ringspot virus* and *Tomato chlorotic spot virus* in vegetables in Florida and the southeastern United States. Phytopathology 105:388–398. doi:10.1094/PHYTO-06-14-0172-R. 25317844

[8] Martinez-Zubiaur Y, Chang Sidorchuk L, González Alvarez H, Barboza Vargas N, González Arias G 2016 First molecular evidence of *Tomato chlorotic spot virus* infecting tomatoes in Cuba. Plant Dis 100:1956. doi:10.1094/PDIS-01-16-0082-PDN.

[9] Adegbola RO, Fulmer AM, Williams B, Brenneman TB, Kemerait RC, Sheard W, Woodward JE, Adkins S, Naidu RA 2016 First report of the natural occurrence of *Tomato chlorotic spot virus* in peanuts in Haiti. Plant Dis 100:1797. doi:10.1094/PDIS-01-16-0070-PDN.

[10] Marshall SH, Adegbola RO, Adkins S, Naidu RA 2017 An efficient and high fidelity method for amplification, cloning and sequencing of complete tospovirus genomic RNA segments. J Virol Methods 242:22–26. doi:10.1016/j.jviromet.2016.12.018. 28082165

